# Smartphone-based quantitative measurements on holographic sensors

**DOI:** 10.1371/journal.pone.0187467

**Published:** 2017-11-15

**Authors:** Gita Khalili Moghaddam, Christopher Robin Lowe

**Affiliations:** Institute of Biotechnology, Department of Chemical Engineering and Biotechnology, Tennis Court Road, University of Cambridge, Cambridge, United Kingdom; Agricultural University of Athens, GREECE

## Abstract

The research reported herein integrates a generic holographic sensor platform and a smartphone-based colour quantification algorithm in order to standardise and improve the determination of the concentration of analytes of interest. The utility of this approach has been exemplified by analysing the replay colour of the captured image of a holographic pH sensor in near real-time. Personalised image encryption followed by a wavelet-based image compression method were applied to secure the image transfer across a bandwidth-limited network to the cloud. The decrypted and decompressed image was processed through four principal steps: Recognition of the hologram in the image with a complex background using a template-based approach, conversion of device-dependent RGB values to device-independent CIEXYZ values using a polynomial model of the camera and computation of the CIEL*a*b* values, use of the colour coordinates of the captured image to segment the image, select the appropriate colour descriptors and, ultimately, locate the region of interest (ROI), i.e. the hologram in this case, and finally, application of a machine learning-based algorithm to correlate the colour coordinates of the ROI to the analyte concentration. Integrating holographic sensors and the colour image processing algorithm potentially offers a cost-effective platform for the remote monitoring of analytes in real time in readily accessible body fluids by minimally trained individuals.

## Introduction

Holographic sensors provide a real-time colour, alphanumeric or image response to the analyte of interest which is readable by the human eye [1–7]. However, the visual inspection of a holographic sensor by an untrained operator is often adequate in providing only semi-quantitative categorical interpretation of analyte concentrations such as positive, negative, high or low. Improving the sensitivity and standardisation of colour quantification of the holographic sensors requires the use of measurement instruments, which in this report, is a camera-enabled mobile phone.

Colour digital imaging is an active research area for quantification in colorimetric instruments. The scope of the work reported in the literature is diverse and covers a wide range of sensors and digital image-based colour analysers. However, standard colour image processing techniques [8–15] are not applicable to holographic reflection sensors, which have an intrinsic reflectivity, are dependent on the illuminating light intensity and exhibit a replay wavelength which depends on the angle of view and which may or may not change with sample application. Accordingly, the captured image of a holographic sensor can lead to an image with a complex background, including highlights and shadows, which demands an improved and sophisticated colour image processing algorithm to recognise automatically the region of interest (ROI), i.e. the colour response of the holographic sensor.

The first step of digital image processing for such heterogeneous systems is image segmentation that partitions the image into regions, each of which represents a homogenous region with respect to selected features. In most of the previous studies, semi-automatic methods were considered, which requires a user intervention to select manually the ROI based on subjective evaluation. Such repetitive manual image segmentation is time-consuming and is not justifiable for the efficient management of the substantial volume of information obtained from multiple sensors. Thus, it would be useful if the segmentation task is automated. Garcia *et al*. [10] highlighted the possibility of automatic image segmentation using an edge detection algorithm to locate discontinuities in images; however, this method was applied to images which were captured under controlled conditions in front of a simple, homogenous background that may not be applicable in real-world situations. The second step of image processing is descriptor selection to present each segment based on its colour characteristics. Accordingly, the selection of an appropriate colour model (colour space) is critical. Several colour spaces have been used in the literature because the selection of the colour model depends on its application and it is not possible to develop a universal approach. Therefore, for any given application, it is necessary to evaluate the performance efficiency of various combinations of absolute and hybrid colour descriptors. The third step of colour image processing is object recognition to identify the ROI. Byrne *et al*. [13] employed a matching algorithm for automatic, unsupervised ROI recognition; however, this method has a low degree of tolerance to variations in the patterns to be matched and becomes time-consuming for complex images in real-world applications.

Previous studies in the field of digital image-based colorimetry [8–15] used different predictive modelling (regression analysis) methods to investigate the relationship between the analyte of interest and the colour response. Simple statistical models have been most commonly used; however, the superiority of neural networks in modelling complex nonlinear systems and increasing the prediction accuracy has been demonstrated and achieved by incorporating a multi-dimensional vector of dependent variables as the input of the network and setting nonlinear equations between them using weighting parameters. It should be emphasised that a predictive modelling method is a function of the sensor type and the engineering behind its design, and thus, for any given sensor platform, it is required to develop a sensor-specific model.

Colour image processing algorithms can be segregated into both system- and software-levels. At the system-level, the camera reproduction of a colour of the original object is a function of its spectral sensitivity profile, which is utilised as a weighting function to determine the RGB values. Such colour rendering is device-dependent. Most of the previous studies inputted the RGB values directly into the software-level and hence developed device-dependent image processing algorithms which do not allow accurate generalisation of the algorithm with different devices. Therefore, it is preferable to develop a device-independent algorithm at the software-level. Device-independent colour reproduction is a systematically formulated colour imaging model that provides a single, standard representation of colour. However, the spectral sensitivity of a digital camera is not a linear transform of the CIE colour matching functions [16], mainly due to the need for maximising the signal-to-noise ratio, and thus the device-dependent RGB values are not linearly related to the device-independent values. Shen *et al*. [8] converted RGB values to the device-independent colour space of CIEL*a*b* using CIE1931 standard conversion metrics on the assumption that this was an ideal imaging device with spectral sensitive profiles equivalent to the CIE1931 RGB colour matching functions. However, this creates a conversion error, and to obviate this, it is essential to characterise the camera and develop an appropriate camera model to convert the RGB values to a device-independent colour space and, eventually, a device-independent algorithm at the system level. Ideally, a generic camera model would allow the development of a device-independent algorithm at both system and software levels.

Thus, digital image processing for holographic sensors requires the development of specific algorithms for colour quantification in which the camera characterisation process is incorporated. Moreover, the requirements for operation in real-world applications whilst largely ignored in previous studies [8–15], should also be considered. An automatic colour processing algorithm is essential to identify the sensor in a captured image where the colour coordinates of the background objects might be similar to the operational colour range of the sensor. A reliable transfer of image data from the smartphone of the user to a secure cloud demands image data encryption, while image compression is required to allow transporting the image data with a low bandwidth in limited-resource settings.

**Fig 1:**
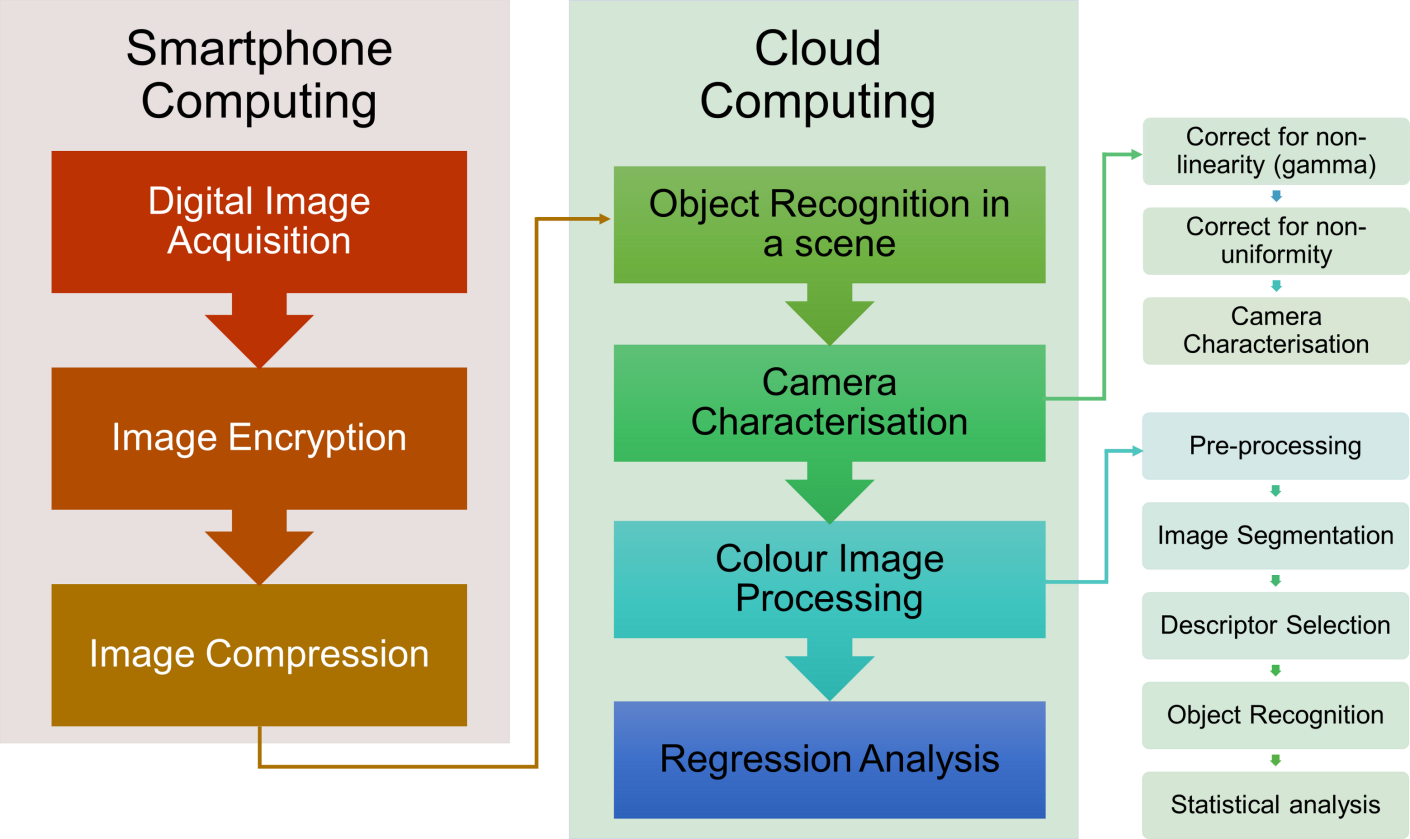
Flowchart of colour quantification based on digital colour image processing for holographic grating sensors.

This report shows that colour quantification can be achieved when the user locates, identifies and captures the image of the holographic sensor on a mobile phone, uploads the image to an app, which automatically executes the encryption and compression algorithms and subsequently, transfers the output image to the cloud for further automatic processing and colour quantification (Fig 1).

The feasibility and limitations of developing a smartphone-based hologram reader is exemplified for the response of pH-sensitive holographic sensors to various pH buffer solutions in controlled conditions where the ambient illumination and the camera characteristics were known and the distance and the angle between the smartphone and the sensor were fixed.

## Methods and materials

The smart polymer was synthesised following a standard protocol in the Institute of Biotechnology, University of Cambridge, UK. In order to covalently immobilise the hydrogel, the glass slide was modified with 2% (*v*/*v*) solution of 3-(trimethoxysilyl) propyl methacrylate in acetone and dried overnight in the dark at room temperature (20–23°C). Subsequently, the modified glass slide was rinsed with ethanol and dried at room temperature.

UV-initiated bulk polymerisation was used for the preparation of the smart hydrogel. The monomer phase, including 91.5*mol%* HEMA, 2.5*mol%* EDMA, 6*mol%* MAA, was dissolved in DMSO containing 2% (*w*/*v*) DMPA. The monomer mixture was vortexed for 5*min* to effect dissolution. HEMA forms the backbone polymer chain and EDMA creates junctions for a cross-linked network. The low percentage of the cross-linker leads to a relatively soft polymer with a greater degree of swelling which in turn results in relatively larger Bragg shifts in response to changes in analyte concentration. MAA is the functional monomer that makes the polymer sensitive to pH changes by providing an ionisable carboxyl group. DMPA is a radical generator to initiate the polymerisation reaction.

The mixture was degassed by sonication for 10*min*. A 200μ*l* droplet of the monomer mixture was pipetted onto the aluminium side of an aluminised polymer film and a silane-treated glass slide was then gently lowered onto the monomer mixture (modified side down). The monomer volume has been experimentally optimised in the Lowe group to obtain the minimum variability across samples. The smart polymer was synthesised via UV polymerisation at 365*nm* for 25*min*. Afterwards, the glass slide was removed from the aluminium surface and washed with ethanol to remove unreactive monomers and DMSO. Subsequently, the polymer was dried at room temperature.

The holographic sensors were developed by the photochemical method [1]. The entire process was carried out under a red safe light. A droplet of 200μ*l* 0.25*M* silver nitrate was applied on a carrier glass and the glass slide was placed on the solution, polymer side down, for 2*min*. Subsequently, the excess solution was wiped off with a clean tissue. The polymer was dried with a cold blower to facilitate the diffusion of the following reactants into the hydrogel. Afterwards, the slide was immersed face-up in a solution of 40*ml* 0.25*M* sodium bromide and 2*ml* 0.5% (*w*/*v*) QBS dye in 3:2 (*v*/*v*) methanol/water under agitation for 45*s*. This timing was experimentally optimised for the grain size of silver bromide to achieve maximum resolution and minimum scattering phenomena. Afterwards, the polymer film was washed copiously with deionized water to remove excess silver bromide. The glass slide was exposed to two pulses at 532*nm* with a Q-switch setting of 275μ*s*. In the highly illuminated regions, the rate of reduction increases and bright fringes with a higher refractive index are formed, whereas in the intensity minima, the reduction occurs less strongly, resulting in dark fringes. After the laser exposure, the polymer film was washed briefly with deionized water and immersed face-up in the developer bath under agitation for 10*s*, containing Saxby A: Saxby B 1:1 (*v*/*v*), where Saxby A is 3*g* metol and 20*g* ascorbic acid in 500*ml* deionized water and Saxby B is 50*g* sodium carbonate and 15*g* sodium hydroxide in 500*ml* deionized water. Afterwards, the polymer was immersed in the 1% (*w*/*v*) sodium bisulphate (Stop bath) for 1*min* to prevent the growth of silver nanoparticles which could reduce the spatial resolution of the grating. Then, to remove undeveloped silver bromide in dark fringes, the glass slide was placed in 12% (*w*/*v*) sodium sulphite (Hypo solution) for 10*min*. The sulphite creates a strong complex with undeveloped silver and removes it from the hydrogel which leads to shrinkage and hence a blue shift. Removing undeveloped silver bromide frees the attached dye in the hydrogel network which can be easily removed in an ethanol: water 1:1 (*v*/*v*) (dye washer) bath. The last two steps improve the brightness of the holographic grating by removing unreacted reactants and thus increase the difference in the refractive indices of the bright and dark fringes. Afterwards, the glass slide was immersed in the bleach solution, 2% (*w*/*v*) copper sulphate and 0.4% (*w*/*v*) potassium bromide in deionized water, for 15*s* to transfer the amplitude grating to a phase grating by converting metallic silver to silver bromide; at this stage, the hydrogel film becomes colourless.

The colour response of the pH-sensitive holographic sensor was evaluated across buffers ranging in pH between 3.00 and 6.50. The recipes for each buffer was taken from the website: https://www.liverpool.ac.uk/buffers/buffercalc.html. The following buffer species at a concentration of 10*mM* were used: Phosphate (pH 3 and 6.20–8.20), acetate (pH 4.00–5.00) and Bis-Tris (pH 5.00–6.20). The pH was adjusted by dropwise addition of hydrochloric acid or sodium hydroxide. The pH buffer solutions used for this work were 3.00, 4.00, 4.50, 4.75, 5.00, 5.25, 5.50, 5.75, 6.00, 6.25 and 6.50.

The acquisition of true colour images from the holographic sensor was performed under controlled conditions in response to pH buffer solutions. The hologram was integrated into a logo matrix barcode (QR code) with a black central space and the pH buffer solutions were applied. The required information about the sensor was incorporated the encoding region. The diffracted colour was captured using a smartphone (Samsung GT-S5660). As the baseline for the studies described in this report, the smartphone was placed in a fixed position at a distance 20*cm* from the sample with a 25°±1.5° viewing angle. The effect of the viewing angle on the perceived colour was previously investigated in the Lowe group [2] and an angular tolerance of ±1.5° was demonstrated for a reproducible measurement. The image database was composed of 66 true colour images [3] of six pH-sensitive holographic gratings sensors in pH buffer solutions (11 pH values between 3.00 and 6.50). The pH sensors were obtained from six different batches. Since the preliminary studies demonstrated a strong agreement between the colour features of 3 to 5 consecutive images of the sensors, one image was captured from each sensor unless the image was blurry [4] due to ambient vibrations. The images had a size of 2048 by 1536 pixels.

The overall architecture of the image encryption technique involved confusion and diffusion steps [5, 6]. In this work, an algorithm to generate the carrier image using a unique passcode was implemented for personalised image encryption. Each character of the personalised alphanumeric password was encoded, and consequently, the carrier image was generated. For passwords in English, 36 binary codes were required to cover 26 letters and 0–9 figures. Once the binary codes of each character were converted to decimal, the permutation matrix was generated and the input image was highly encrypted.

Single-level two-dimensional wavelet-based image compression was applied [7–9] using the Wavelet Toolbox of MATLAB to achieve temporal and spatial localisation simultaneously. The performance of Haar and Daubechies wavelets was evaluated.

A decrypted and decompressed image was obtained by applying the reverse processes and then the sensor was identified in the captured image. An object recognition algorithm based on template matching was used to locate the QR code with the embedded sensor in the scene. The Compute Vision Toolbox of MATLAB was used to apply the Speeded Up Robust Features (SURF) [10] method for the feature detection. The target image of the scene was converted into the greyscale before finding point correspondences between the template and target (the image of the scene) images. This algorithm can detect the QR code despite a scale, in-plane rotation and a low degree of out-of-plan rotation.

The next stage of the algorithm is camera characterisation which refers to converting the device-dependent colour (RGB) to a device-independent colour model such as CIEXYZ. The homogeneity in the lighting of the captured image is critical for camera characterisation. To assure repeatability of the image acquisition and uniformity of the lighting, the non-linear response of camera RGB channels was investigated under laboratory-controlled illumination (CIED65 of a light booth) and viewing conditions using a colour chart with known device-independent coordinates (ColorChecker® Classic). The elapsed time of the light booth was recorded to ensure the illuminance of the MiniMatcher light booth is maintained according to its operational specifications. Despite using a light booth, the uniformity in the lighting of captured images is not attainable practically. This is due to the sensor non-linearity and non-uniformity, which needs to be addressed prior to camera characterisation.

The non-linearity of the imaging sensor in capturing luminance [11] was corrected using the gamma correction algorithm suggested by Sharma and Bala [12], where the relationship between incident radiance and camera response for the 6 grey scale patches of ColorChecker was determined. The RGB values of the individual grey patches were mapped to the corresponding normalised luminance values and then, the gamma value of each RGB channel was calculated. The spatial non-uniformity which is caused by various noise sources [13] and the falloff in illumination at the edges of the image due to lens vignetting [14] were corrected using a scene-based technique suggested by Hardeberg [15].

Once the ColorChecker image was corrected for non-linearity and non-uniformity, the mean RGB values of each colour patch were extracted. The XYZ tristimulus values for each 24 colour patch were calculated using their spectral reflectance 380-780nm in 5nm intervals under illuminant CIED65 and the 2° CIE1931 standard colorimetric observer and illuminant CIED65. The spectral reflectance of colour patches was obtained from the webpage of Rochester Institute of Technology [16]. The CIE1931 colour matching functions were obtained from the UCL Institute of Ophthalmology webpage [17]. Afterwards, the CIEXYZ values were converted to CIEL*a*b* [18] to enable quantification of the performance of camera characterisation methods. The tristimulus of the reference illuminant was considered CIED65 white light. Polynomial modelling of 24 patches of the ColorChecker was used to derive a matrix to transfer the camera RGB outputs to XYZ values [14,19]. The suggested augmented RGB terms were based on a study by Cheung and Westland [20]. Once the coefficients of polynomial models were estimated, the CIEXYZ values of the 24 colour patches of the Colorchecker were calculated. To evaluate the performance of various orders of polynomial models, the mean of resulting XYZ tristimulus values were converted to CIEL*a*b* values, and subsequently, the colour difference, Δ*E*_*ab*_, between the reference and the calculated CIEL*a*b* values for each polynomial was calculated [18, 21–23]. Afterwards, the median of Δ*E*_*ab*_ for various degrees of polynomial models was obtained.

The colour image processing stage of the algorithm includes three key steps of image segmentation, descriptor selection and object recognition after the pre-processing step to derive the device-independent colour values. The purpose of this stage is to extract the region of interest (ROI), i.e. the colour response of the hologram, from the captured images. The captured images of the colour response of holographic grating sensors to pH buffer solutions were pre-processed using the camera characterisation algorithm, where the images were initially corrected for non-linearity and non-uniformity and subsequently, the polynomial model of the camera with 22 RGB terms was applied to compute the corresponding XYZ tristimulus. Afterwards, the CIEXYZ matrix was reshaped to a two-dimensional matrix and the CIEL*a*b* values were calculated. The extracted RGB values were also used to calculate the HSI colour values.

Image segmentation is the process of partitioning the image into distinct regions, each of which represents a homogeneous region with respect to selected features. Prior to the image segmentation, a two-dimensional (2D) Gaussian-based smoothing filter [24] was applied to reduce the noise. Since there is no clear standard to determine which segmentation technique should be used for an image, the candidate image segmentation techniques were selected based on the pixel clouds of the captured images and their performance was then evaluated. The pixel clouds were visualised in the RGB, HSI and L*a*b* colour spaces and subsequently, two pixel-based clustering methods, including k-means [25] and fuzzy c-means (FCM) [26] were used; the latter is the fuzzified version of the k-means. Three colour spaces of RGB, HSI and CIEL*a*b* were considered for these segmentation methods. These segmentation methods are unsupervised and hence a quantitative metric is required to control the number of compact clusters (segments) that are well separated. In this report, a validity measure (*VM*) proposed by Turi and Ray [27] was applied to optimise the number of clusters:

$$VM=y\left(k\right)\frac{intra}{inter}$$ (1a)

where *intra* is a measure of cluster compactness, *inter* is a measure of distance between the clusters and *y* is a function of number of clusters:

$$intra=\frac{1}{M}\sum_{i=1}^{c}\sum_{x\in C_{i}}{\left\|x-c_{i}\right\|}^{2}$$ (1b)

$$\forall i=1,2,\ldots ,c-1,\forall j=i+1,\ldots ,c\;inter=min({\left\|c_{i}-c_{j}\right\|}^{2})$$ (1c)

y=mN(μ.σ)+1 (1d)

where *M* is the number of pixels in the image, *C* is the number of clusters and *c*_*i*_ is the colour of the cluster centre *C*_*i*_. The squared Euclidian distance increases the importance of large distances while weakening the importance of small distances. The multiplier function *y* is the Gaussian function of the number of clusters with a mean of μ and standard deviation of *σ*:

$$N(\mu .\sigma )=-\frac{1}{\sqrt{2\pi \sigma^{2}}}e^{\left[-\frac{{\left(k-\mu \right)}^{2}}{2\sigma^{2}}\right]}.$$ (2)

Given the complexity of the background, including highlights and shadows, more than two segments of background and foreground were required to represent adequately variations in colour coordinates of the image, and hence, the mean μ was set at 2. The standard deviation *σ* was set at 1 to avoid over-segmentation and affecting the number of clusters between 2 and 5 in accordance with the empirical rule [28] that 99.7% of values fall within the range of μ*+*3*σ*, which equals to 5 segments. The constant *m* was set at 20, which has been shown [27] to be a minimum value that appropriately segments various types of images. The *VM* minimization was obtained for the optimal value of the number of clusters (*k*) for each segmentation method. The constant 1 in Equation 1d maintains the original ratio of *intra* to *inter* where the Gaussian function is not effective in large numbers of clusters (*k*). This algorithm searched for the minimum value of *VM* for the number of segments between 2 and 10.

The image segmentation step provides raw data in the form of distinct regions. The next step is descriptor selection to represent and describe the regions in characteristic features for further processing. In this study, an individual region in the captured colour image was presented based on colour. However, the selection of an appropriate colour model (colour space) is critical. To select the most suitable colour space for a hologram identification approach, the performance of the colour spaces was evaluated in terms of separability of various classes in different colour spaces of RGB, HSI and CIEL*a*b* [29]. The quantitative metric *J* was measured based on intra-class (*S*_*w*_) and inter-class (*S*_*b*_) covariance matrices:

$$J=trace(\frac{S_{b}}{S_{w}}).$$ (3)

The intra-class matrix (*S*_*w*_) indicates the distribution of data (*X*_*n*_) around their respective mean of the class and is defined as:

$$S_{w}=\sum_{i=1}^{c}S_{i}$$ (4a)

$$S_{i}=\sum_{n\in C_{i}}(X_{n}-M_{i}){\left(X_{n}-M_{i}\right)}^{T}$$ (4b)

$$M_{i}=\frac{1}{N}\sum_{n\in C_{i}}X_{n}$$ (4c)

$$N=\sum_{i}N_{i}$$ (4d)

where *c* refers to the number of classes, *M*_*i*_ is the mean of class, *C*_*i*_, *N*_*i*_ is the number of members in class *C*_*i*_ and *T* represents the matrix transpose. The inter-class matrix (*S*_*b*_) represents the distribution of samples around *M*, the mean of the means of the classes (grand mean):

$$S_{b}=\sum_{i=1}^{C}N_{i}\left(M_{i}-M\right){\left(M_{i}-M\right)}^{T}\;\mathrm{and}\;M=\frac{1}{N}\sum_{n=1}^{C}N_{i}M_{i}.$$ (5)

The next step of the colour image processing stage is object recognition to determine the class (label) of objects. An unsupervised neural network, self-organising map (SOM) of 10 by 10 nodes [30, 31], was used for classification and recognising the segment of the ROI in the segmented images using the selected descriptor values.

In this paper, two segmentation methods (k-means and FCM) were applied to the image dataset. The evaluation of the degree of consistency between these methods requires evaluating the segmentation algorithms based on the performance of the colour image processing stage where the ROI is recognised. The suggested metrics were applied to the mean value of colour features of ROI in the RGB, HSI and CIEL*a*b* colour spaces. A common metric for quantitative analysis of the segmentation methods is the intra-class correlation coefficient (*ICC*) [32] which represents the consistency between segmentation methods:

$$ICC=\frac{{MS}_{B}-{MS}_{W}}{{MS}_{B}+(k-1){MS}_{W}}$$ (6)

and the between-targets mean squares *MS*_*B*_ and the within-targets mean squares *MS*_*W*_ are defined as:

$${MS}_{B}=\frac{1}{n-1}\sum_{x}{k(m_{x}-\mu )}^{2}$$ (7a)

$${MS}_{w}=\frac{1}{n(k-1)}\sum_{x}{\left(x-m_{x}\right)}^{2}$$ (7b)

where *n* is the number of targets and *k* is the number of segmentation methods which are respectively 3 and 2 in the case of comparing the three colour features of ROI of two segmentation methods, *m*_*x*_ is the mean of all segmentation methods on target *x* and *µ* is the grand mean. In this paper, the three colour coordinates of ROI for each colour space are the targets for colour quantisation by *k* segmentation algorithms on each image set. The value of *ICC* will be increased where there is a high correlation between the image segmentation methods which demonstrates inter-method reliability. Moreover, the Bland and Altman method [33] was used to establish the degree of concordance between the pair of segmentation methods and to investigate the interchangeability of two methods without assuming that either is the gold standard.

Regression analysis is the final stage of the algorithm which is targeted at predicting the concentration of an analyte by means of colour digital imaging by investigating the relationship between descriptors and analyte concentration. Once this relationship is established, the calibration method is used to estimate an unknown analyte concentration from the response of a custom-developed smartphone-based reader. The colour features of the ROI, in this case the hologram, were obtained in the RGB, HSI and CIEL*a*b* colour spaces. The colour features were visualised to explore whether the absolute colour value in the proposed colour spaces has the ability to represent the response of the hologram across a wide range of pH values (3.00–6.50). Accordingly, a hybrid combination of colour coordinates was considered as the feature vector of the ROI. Afterwards, a multilayer perceptron (MLP) [34, 35] neural network was used to learn the relationship between the colour descriptors and the analyte concentration, which are sensor-specific. The MLP had three layers, 2 hidden layers and one output layer, with 9 input nodes. Each node of the hidden layer had a log-sigmoidal activation function and the output layer had a linear transform function. The maximum iteration number was set at 10K and an error limit of 1E-6 was assigned. The image database was divided into training and test data sets, respectively 44 (4 sensors; 11 pH levels) and 22 (2 sensors; 11 pH levels) images. The test data was used to simulate future data points in evaluating the accuracy of the network. The network was trained based on the backpropagation rule using the colour descriptors. The MLP performance was assessed in terms of the accuracy of estimation of the analyte concentration.

In terms of materials, all reagents **were purchased from Sigma-Aldrich Chemical Company Ltd (UK) in analytical grade unless otherwise stated. Chemicals for silanization include 3-(trimethoxysilyl) propyl methacrylate and acetone, whilst for the hydrogel synthesis they include 2-**hydroxyethyl methacrylate (≥99.9%; HEMA) as the backbone monomer, methacrylic acid (99%; MAA) as the functional monomer, ethylene glycol dimethacrylate (98%; EDMA) as the crosslinking agent and 2,2’-dimethoxy-2-phenylacetophenone (99%; DMPA) as the photo-initiator; the polymerisation was performed in dimethyl sulphoxide (≥99.9%; DMSO) as solvent. The required reagents for the development of the poly(HEMA) holographic grating sensor were silver nitrate (≥99.9%), 1,1’-diethyl-2,2’-cyanine iodide (97%; QBS dye), sodium bromide (≥99%), ascorbic acid (≥99%), sodium carbonate anhydrous, sodium hydroxide, sodium bisulphate, ethanol (~95%) and copper sulphate (≥99.99%). Chemicals for pH buffer solutions include phosphoric acid, sodium phosphate monobasic (≥99%) and acetic acid (≥99%). Chemicals that were supplied by Acros included 4-(methylamino) phenol hemisulphate salt, ≥98% (Metol) and potassium bromide (≥99%). Methanol (≥99.8%) **and** sodium sulphite (98.5%) were **purchased from Fisher Scientific (UK). Freshly distilled and deionized water was used to prepare all solutions.**

Microscope slides (1.0–1.2*mm* thick) were purchased from Fisherbrand™. Aluminised polymer films with a thickness of 125μ*m* (MEX12C) were purchased from HiFi Industrial Film Ltd (UK). The UV exposure unit was an EPROM Eraser supplied by Electroplan.

A standard bench-top pH meter (Accumet™ Basic AB15, ±0.01 pH unit), electrode and calibration buffers were purchased from Fisher Scientific Ltd, UK. A frequency-doubled Nd:YAG (20*w*, 2*J*, 10*Hz*, 532*nm*, Brilliant B, Quantel, France) was used for the holographic grating development. The fibre optic cables (FC-UV200; 200μ*m* core diameter; single fibre; 1-2*m* long) were purchased from Avantes. Unless otherwise stated, images of the sensor were captured under a controlled illumination setup of the artificial daylight (CIED65) using a compact colour matching booth (GTI Minimatcher MM-1e/65). For the spatial non-uniformity correction, the ColorChecker® 18% Grey Balance target (101*mm*×178*mm*) was purchased from X-Rite (Macbeth). The reference test colours for camera characterisation were obtained from the X-Rite (Macbeth) ColorChecker® Classic (21.59*cm*×27.94*cm*) with 24 patches to provide a suitable representation of colours [36] including six neutral, red-green-blue (RGB), cyan-magenta-yellow colours. The 18% grey reference is also used in the standard 24-patch ColorChecker. The digital images, which are associated with the hologram response to the test solutions, were captured using a Samsung smartphone, model GT-S5660 (3.15M Pixel). The camera phone was set to automatic focus, white balance, sensitivity, centre weighted and captured in single-shot mode. These are the standard conditions that are expected any user to consider. Each image was recorded as a JPEG (24 bits) on a Kingston microSD card. The images were transferred to a desktop computer (64 bit Windows 7 Professional, Intel® Core™ i5-2500K CPU @ 3.30GHz, RAM 8GB) for subsequent processing. The colour image processing algorithm was developed in MATLAB® R2012a.

## Results

**Fig 2:**
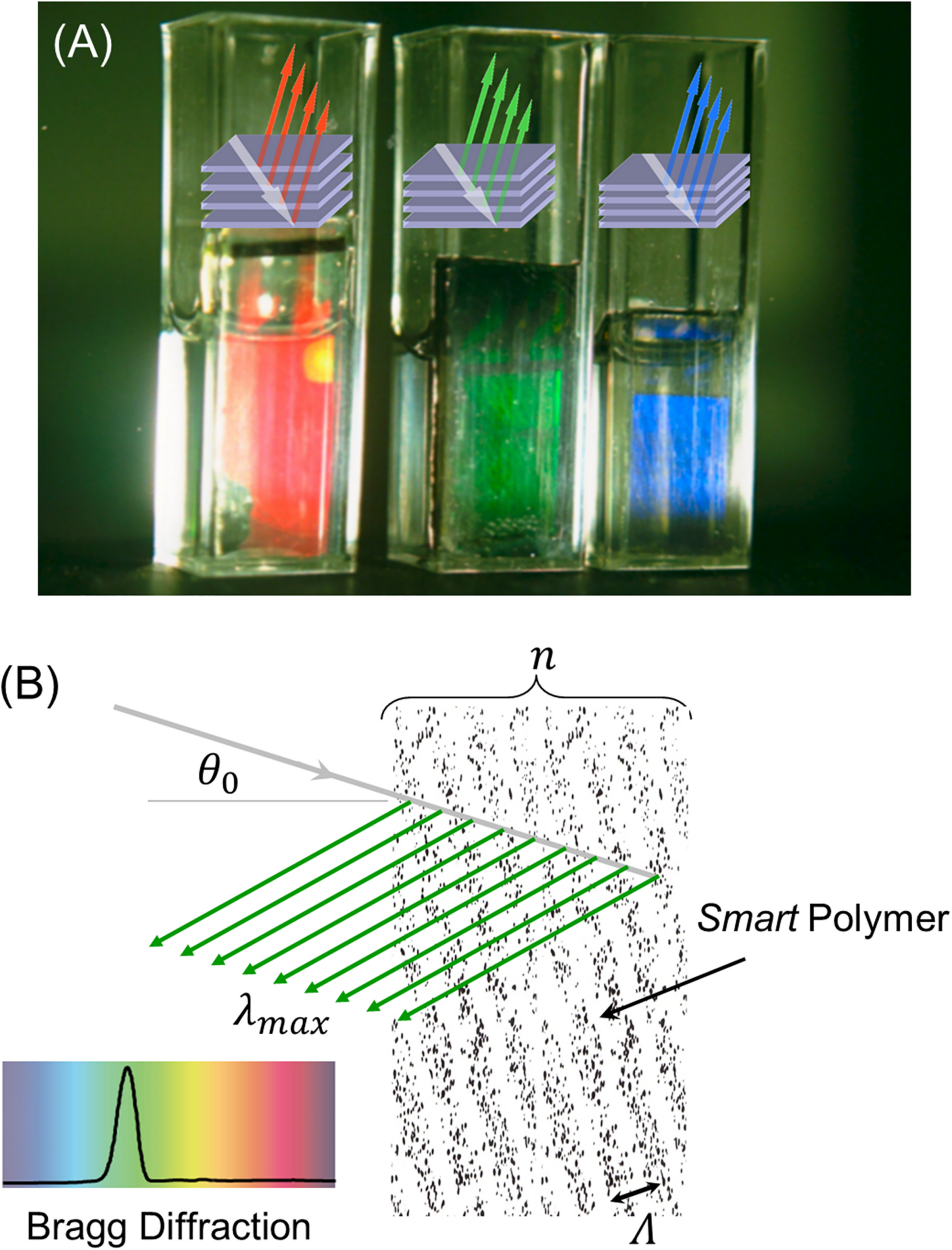
Tuning structural colour in a pH-sensitive holographic sensor developed in the Lowe group. (A) Structural colours in three different pH solutions of 6, 5.25 and 4.75, from left to right; (B) The colour is tuned by controlling the parameter *d*, the grating spacing and consequently Bragg diffraction from the holographic grating.

The mechanism of colour change in holographic sensors is driven by the kinetics of volume changes in the smart hydrogel [37]. The colour of the smart pH hydrogel used in the work described in this paper reversibly changes from blue to green to red in response to changing proton concentrations. The reversible swelling/deswelling characteristics of a pH-sensitive hydrogel are defined by the chemistry of the polymer film. In HEMA-MAA copolymers, the swelling equilibrium is a strong function of the functional monomer (MAA). At the apparent pK_a_ value of the MAA-containing hydrogel (6.01), there is an equilibrium between co-existing deprotonated (–COO^-^) and protonated (–COOH) forms of the carboxyl group of MAA. This equilibrium tends towards the deprotonated form at pH values above the pK_a_ value. Charge neutrality is maintained by cations that enter the hydrogel with conjugated OH^-^. The increased cation concentration in the hydrogel leads to an osmotic pressure gradient that causes the polymer film to swell. Additionally, the deprotonated carboxyl group is more hydrophilic than the protonated one which leads to absorbing more water and further expansion. Therefore, starting from low pH values, the hologram swells following a sigmoidal profile centred at the apparent pK_a_ value of the polymer, which is typically one unit less than that of the functional monomer due to proximity effects and intra-network interactions. Fig 2 illustrates the response of a carboxyl-functionalised acrylic polymer pH-sensitive sensor to pH changes in the range 4.75 to 6.00, resulting in the swelling of the smart hydrogel and, ultimately, leading to a red shift in the diffraction spectrum. The degree of volume change and hence wavelength shift is found to be a function of the number of covalently attached charged groups in the hydrogel matrix.

**Fig 3:**
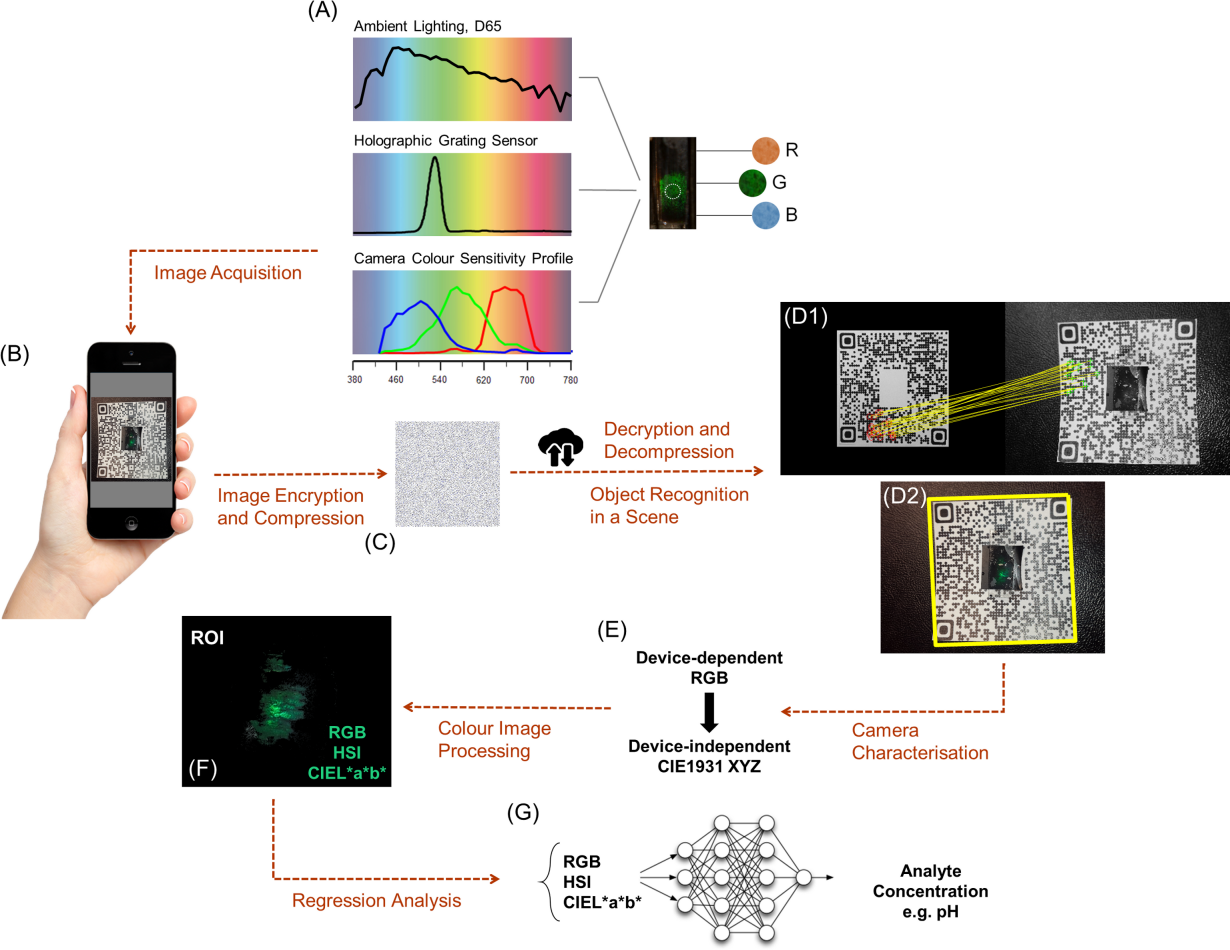
(A) Image acquisition: The captured colour is a function of ambient illuminant (D65 in this study), the replayed colour of the holographic sensor and the spectral sensitivity (characteristics) of the camera (Samsung GT-S5660); (B) An example of captured image of the holographic sensor in the centre of a logo QR code (encoded data: The Lowe Group, Institute of Biotechnology, University of Cambridge); (C) Encrypted and compressed image uploaded to the cloud, followed by decryption and decompression for further image processing; (D) Object recognition in a scene using the SURF method: (D1) Matched the strongest feature points (yellow lines) from the template image in the greyscale to an in-plane rotated image (outlined in green); (D2) Detected QR code with the embedded sensor in the scene (outlined in yellow); (E) Camera characterisation to convert the device-dependent RGB colour values to device-independent CIEXYZ tristimulus values; (F) The corresponding region of interest (ROI); (G) Regression analysis using a multilayer perceptron to derive the analyte of concentration (e.g. pH).

The colour response of holographic sensors is readable by human eye; however, there are limitations for colour discrimination due to the inherent limitations of human colour vision, colour memory loss, eye fatigue and colour blindness. Accordingly, standardisation of colour quantification by instrumentation is essential. As shown in Fig 3A and 3B, the captured colour by the camera of the smartphone is a function of the ambient illuminant, the camera characteristics and the replayed colour of the sensor in the response to the analyte concentration. In this set of experiments, knowledge about the ambient illuminant was available (artificial daylight; D65) and the camera colour sensitivity profiles were extracted through the camera characterisation stage. Accordingly, the colour information of the captured image can be deconvoluted for quantification. Since the proposed colour quantification algorithm is a cloud-based one, image data transformation from the smartphone of the user to the cloud is required. This may raise concerns regarding data security, the overall transportation time and low bandwidths. To address these challenges, the captured image of the sensor was encrypted and compressed to allow its near real-time, secure flow across the network (Fig 3C).

Although a captured image of the sensor *per se* without the colour quantification code may contain less sensitive personal information, this image encryption algorithm still increases the security of image data transition to the cloud, and, in terms of rate of performance, the wavelet transforms performed the task in ~7.7*s* although the Daubechies were on average 0.23*s* slower. The compression rate for the Daubechies and Haar wavelets was 11.4 and 12.6, respectively. Accordingly, the Haar transform slightly outperformed the Daubechies wavelet.

Once the image was securely uploaded on the cloud, the reverse processes were applied to obtain the decrypted and decompressed image. Fig 3D1-2 illustrate object recognition using the SURF method, where the template image of the QR code and the 100 strongest feature points that were selected. These feature points were matched to an in-plane rotated image of the QR code containing the embedded sensor, where the embedded sensor was detected in the overall scene.

The next stage of the algorithm centres around camera characterisation to convert the device-dependent colour features to the device-independent tristimulus values. The captured image of the ColorChecker under illuminant CIED65 was processed to extract the RGB values of the grey patches. The maximum values of red, green and blue channels are 220, 219 and 213 implying that the full 8-bit capacity of the camera is almost entirely exploited. Afterwards, the RGB values of the grey patches were used to determine the nonlinear nature of the camera. The gamma values for each red, green and blue channels were obtained by fitting power functions with degree exponents 1.987, 1.899 and 1.791, respectively. The gamma corrected image was processed for the spatial non-uniformity analysis and the calibration constants of 0.393, 0.408 and 0.421 were obtained for the RGB channels, respectively. Once the camera RGB responses were linearized and the spatial non-uniformity effect was compensated, camera characterisation using the polynomial method was performed. The accuracy of the camera characterisation improved as the number of RGB terms increased and the median Δ*E*_*ab*_ was minimised (0.045) for 22 terms in the transformation matrix which assures precise modelling. Other polynomial models lead to a noticeable colour difference compared with the reference. However, RGB terms above 14 with Δ*E*_*ab*_<4 may provide adequately precise camera characterisation in less demanding commercial applications. Accordingly, the polynomial model with 22 RGB terms was considered to convert the captured RGB values of the holographic sensors in response to test solutions into the independent CIEXYZ tristimulus values which, were subsequently converted to CIEL*a*b*.

**Fig 4:**
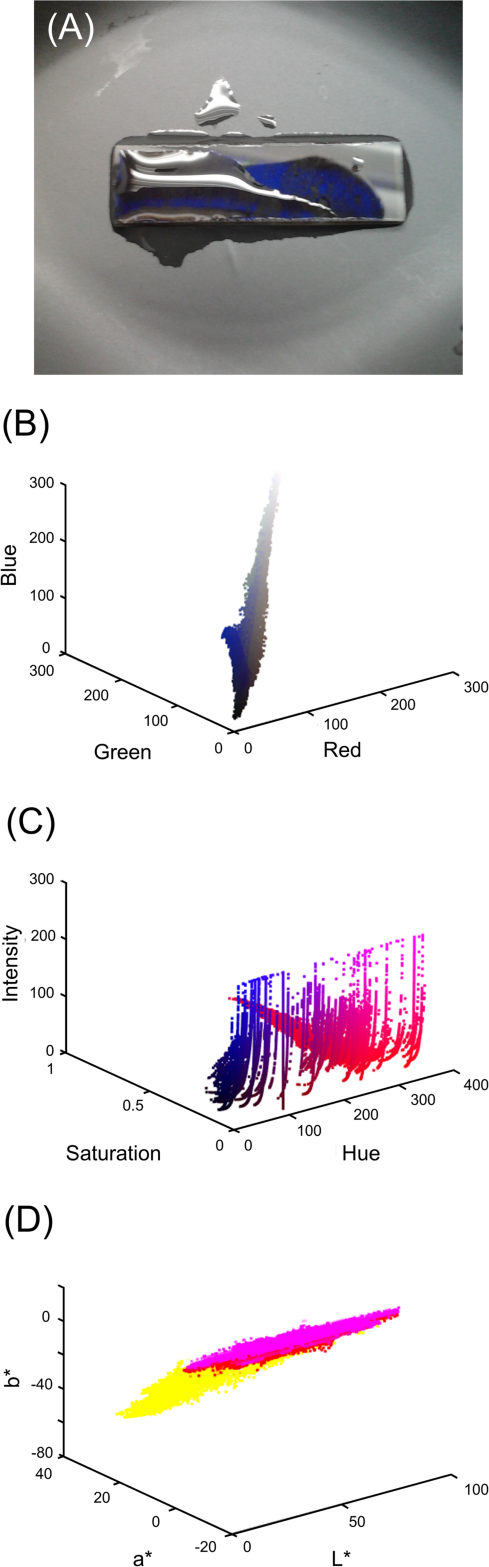
A sample of captured images from the colour of holographic grating sensor at pH 3.00. (A) and the corresponding pixel colour distributions in RGB (B), HSI (C) and CIEL*a*b* (D) colour spaces.

The pixel clouds of one candidate image in RGB, HSI and CIEL*a*b* colour spaces is shown in Fig 4. The clusters of colour features possess a low degree of compactness and separability, which are caused by highlights and shadows. Although there is not an obvious choice of colour space in terms of compactness and separability, the RGB colour space presents a softer separability in comparison with HSI and CIEL*a*b* which the intensity/lightness is separated from the hue coordinate.

The segmentation performance of k-means and FCM clustering methods on the direct image data set was evaluated. The validity measure *VM* was computed for images of the sensor response to buffer solutions in the range of pH 3.00–6.50 and the average for each set of images was driven. The best results in terms of average *VM* were obtained with 5 clusters in the CIEL*a*b colour space for both segmentation methods. The light reflection on the surface of the buffer solution, which was captured as highlights, and also shadows creates various false shades of colours and therefore a colour space that excludes the lightness from the hue such as the CIEL*a*b* outperforms the RGB colour space. The number of clusters was considered 5 for an optimised performance.

Investigations were made to identify a robust colour space to segment the hologram images reliably. Once the colour images were segmented, each image was represented by its corresponding segmented regions and identified by their corresponding colour descriptors. A comparative study was undertaken to select the most suitable colour space for the colour identification approach. Three colour spaces including RGB, HIS and CIEL*a*b* were investigated. The quantitative metric *J* was computed for image data sets within the pH range of 3.00–6.50 and the average value across the pH range described the separability of each colour space for each method (Table 1). Since a higher value of *J* determines the classes are more separated, the HSI and CIEL*a*b colour spaces, which decouple the luminance and chromaticity, are more successful than the RGB model.

**Table 1:** The average of the quantitative metric *J* for three colour spaces of RGB, HSI and CIEL*a*b* for the pair of segmentation method.

Segmentation method	k-mean	FCM
RGB	1.62	1.52
HSI	2.25	2.34
CIEL*a*b*	4.88	4.79

The discriminative classifier, self-organising map (SOM), returned the appropriate label for each object. Classification on the image data set resulted in an accuracy of 93%. The accuracy could be improved up to 96.5%, but at the cost of decreasing specificity. Fig 4 demonstrates an example of implementing the object recognition algorithm on a captured image at pH 3.00.

**Fig 5:**
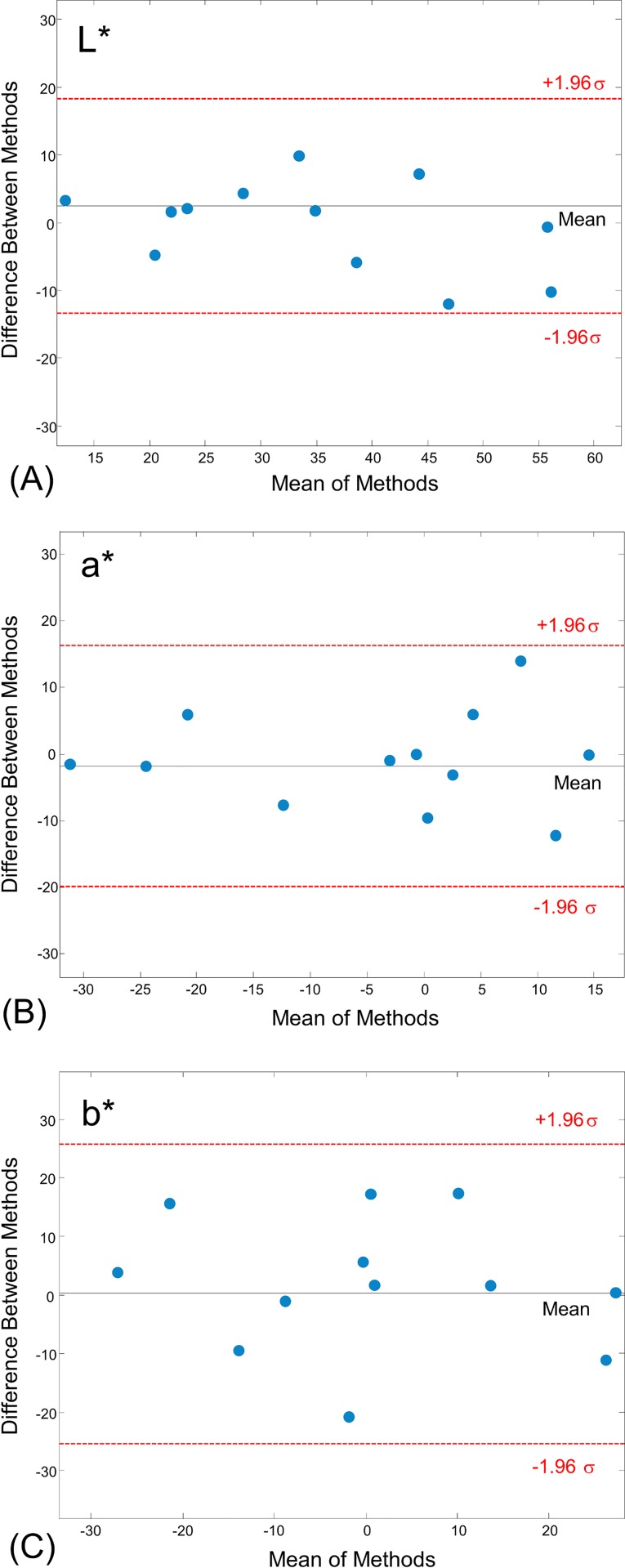
The Bland-Altman plot for the colour coordinates of the CIEL*a*b* colour space for k-means and FCM segmentation methods.

The average intra-class correlation coefficient (*ICC*) across the pH range of 3.00–6.50 was calculated for the ROI of each colour segmentation method in the RGB, HSI and CIEL*a*b* colour spaces. The maximum intra-class correlation in between two segmentation methods of k-means and FCM was for the CIEL*a*b* colour space with the *ICC* value of 0.652 followed by the HSI (0.472) and RGB (0.461) colour spaces. The Bland and Altman plots were used to assess the agreement between two segmentation methods for the pair of the coordinates of the CIEL*a*b* colour space (Fig 5). The mean of the difference between each pair was within the ±1.96σ range that confirms the agreement in between these segmentation methods for this colour space. Since the segmentation methods can be applied interchangeably, k-means, which is computationally less demanding, was selected as the candidate segmentation method.

**Fig 6:**
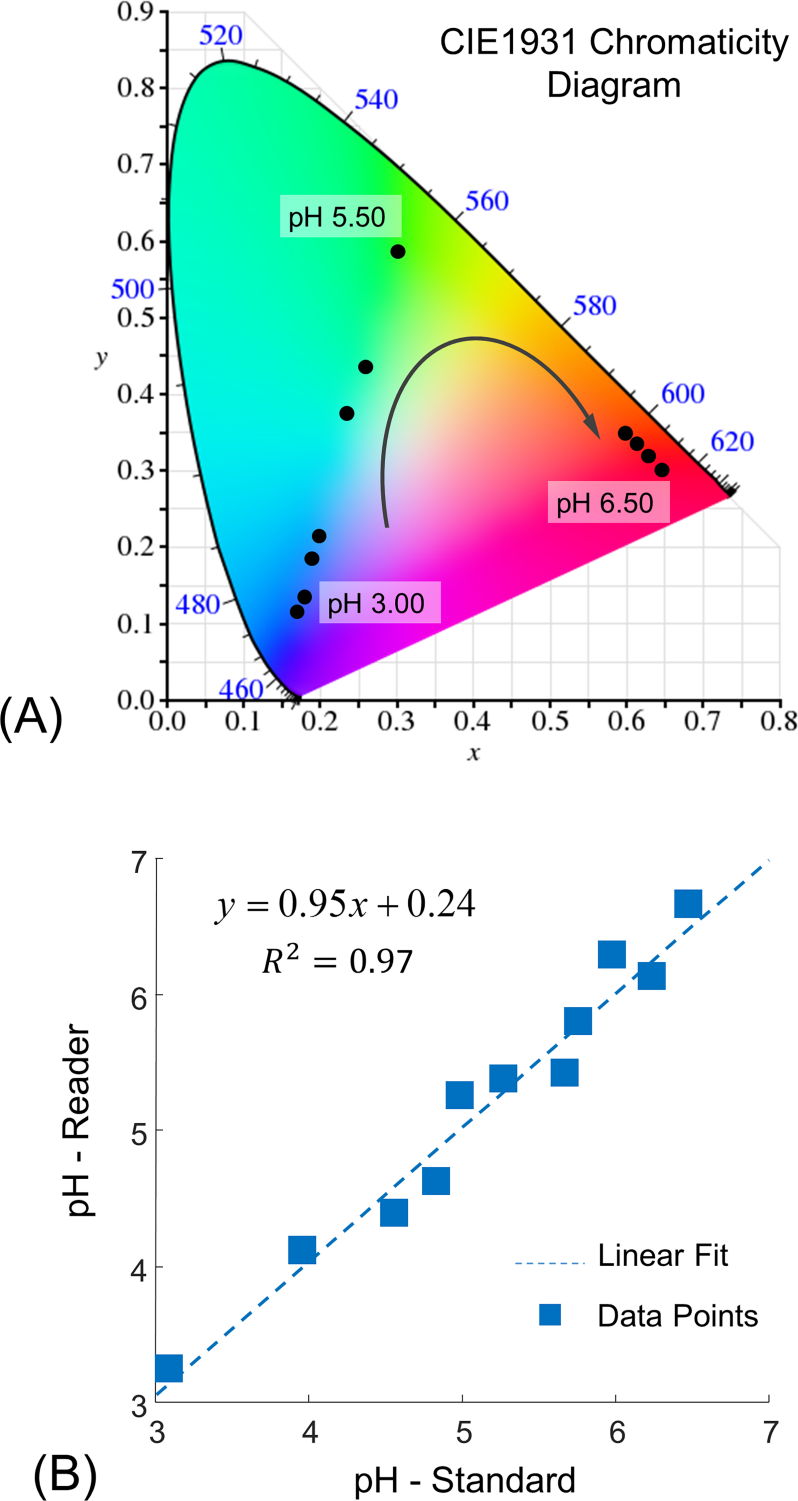
(A) The CIE1931 chromaticity plot for colour coordinates of the pH-sensitive holographic grating sensors to buffer solutions in the pH range 3.00–6.50; (B) The linear correlation between the colour quantification algorithm and the true pH value of the buffer solutions (2 sensors at 11 pH levels).

The mean colour coordinates of the ROI of the image datasets were computed in the CIExy colour space and were displayed on the standard CIE1931 chromaticity diagram as shown in Fig 6A. The colour of the holographic sensors in response to buffer solutions within the pH range 3.00–6.50 varies clockwise from blue to red. Once the optimised neural network was obtained using a database of 44 images, the response of the digital image based colour quantification to changing the pH of buffer solutions in the range of pH 3.00–6.50 was determined for two independent datasets including 2 sensors at 11 pH levels. Fig 6B illustrates the performance of the proposed colour digital image-based colorimetry versus the true pH values of the buffer solutions which is strongly linear.

## Discussion

The proposed digital image based colorimetry achieved nearly perfect colour quantification under controlled conditions (Fig 6). Images of holographic sensors in different pH buffers show quantifiable colour changes in relation to analyte concentration. The digital image based colorimetry was demonstrated to achieve nearly perfect colour quantification in an image database containing images of the colour response of the sensor to pH buffer solutions within the range of 3.00–6.50. This relationship between the response of the algorithm and true pH values was established by the use of a supervised neural network. The threshold of sigmoidal activation functions of the hidden neurones of the network was optimised to minimise the residual standard error, which points to the difference between the actual and predicted values.

Although the algorithms were selected with an understanding of the minimum memory requirements, the computational cost of the proposed algorithm is high, and therefore, a cloud-based system was preferred to maintain the accuracy in the determination of the analyte concentration.

One of the modules that has increased the complexity level of the proposed algorithm in comparison with other studies on digital image-based colorimetry is the camera characterisation module. The accuracy of colour rendering using a digital camera is partially determined by the sensitivity of the image sensor to each red, green and blue colour feature. Accordingly, the captured RGB colour coordinates are device-dependent and this necessitates camera characterisation to derive the camera model. Previous studies used the CIE1931 standard conversion matrices to transfer the colour coordinates between each pair of colour spaces based on the assumption of an ideal imaging device [38–52]. Unlike the existing studies, the proposed algorithm in this study used the camera model to convert the device-dependent RGB coordinates to the CIE1931XYZ colour space. Moreover, an understanding of the un-representable colours of an imaging device is essential to determine the physical limitation imposed by the imaging device because the colour gamut mismatch results in mapping out of gamut colours within the destination space for rendering and, ultimately, introduces a systematic error in the process. This systematic error was observed at the pH range above 5.00 for the imaging device that was used for this work. This is evident from the fact that above pH 5.00, the pH reader recorded close data points for two slightly different pH standard measurements of 0.25 pH units apart. The knowledge of this physical limitation assists with the design of a more readable sensor using commercially available imaging devices embedded in smartphones. Given the variety of commercially available smartphones and their camera characteristics, identifying the limitations of this instrumentation method for a universal camera is required. This challenge will be further investigated in future work.

The response time of the algorithm is another dimension of interest because of its ultimate application for the real-time measurement of clinically important analytes. The optimisation process is challenging because it is necessary to make a trade-off between processing accuracy and computational complexity. For instance, speed enhancement can be achieved by employing relatively inexpensive computational image processing techniques such as low-degree polynomial camera modelling [53], but at the cost of low processing accuracy. Moreover, the performance of each algorithm was evaluated to determine execution of each specific module using the built-in algorithm profiling feature of the MATLAB, while the quantitative evaluation of the overall image processing results assured the accuracy of the performance.

The performance efficiency of the algorithm in different colour spaces was investigated. The HSI and CIEL*a*b* colour spaces are more efficient than the RGB model to separate clusters. However, nonlinear colour models have non-removable singularities [54], whereas the linear RGB space does not have such problems. Instead, the high correlation of the tristimulus components of linear colour models makes them dependent on each other and associate strongly with intensity. To make a trade-off between these two, a hybrid colour space [55], including a combination of colour features, was used in this work to improve the overall performance of the proposed colour image processing techniques.

The cloud-based nature of the proposed colour image processing algorithm demands image data transformation from the smartphone of the user/patient to a secure cloud and returning the results back to the user. This may raise concerns regarding the security and broadband infrastructure requirements. Assuming a secure network, data transition encryption is of particular importance which can be addressed by solutions such as a virtual private network or firewalls to control the access to the service based on the network. These solutions are not applicable in the case of remote monitoring which demands anywhere and anytime access. An alternative approach is data encryption which was explored in this work to provide a reliable transfer of image data across the network to the default cloud of the healthcare provider for colour processing. The image compression step allows transporting the image data with a low bandwidth which is of critical importance in limited-resource settings. Moreover, the image compression algorithm provides an efficient and robust image transfer to the cloud of the healthcare provider by reducing the size and hence the overall transportation time. Accordingly, the demands of a prompt, often real-time, response of this platform as a self-monitoring technique are addressed.

A key step of the proposed digital image-based colour quantification is identifying the sensor in a captured image which is based on a colour cue. Although the colour-based object recognition accurately identified the sensor in the image data sets, this is not necessarily applicable in real-world applications because the colour coordinates of the background objects might be similar to the operational colour range of the sensor. Therefore, automatic sensor recognition in a scene demands incorporating features such as geometrical dimensions and pattern into the object recognition algorithm. A pattern-based technique using a QR code was explored in this work. However, the platform is still accessory-free because the QR code will be incorporated into the design of the holographic grating sensor substrates for the blood and urine samples. The information of the QR code can be personalised for the patient and therefore, the captured image of the sensor would provide also a unique identification number which facilitates the electronic health record. The object recognition algorithm for other forms of the holographic sensors merely will use the colour cue, such as the skin colour cue for smart tattoos, or the geometrical dimensions.

## Conclusions

This work has confirmed the feasibility of integrating a smartphone-based instrumentation method with the holographic sensor platform. Holographic sensors can be utilised to detect chemicals and biomarkers by tuning the properties of the functionalised matrix (analyte receptor) and the holographic grating (refractive index and grating spacing) and consequently the location of the spectral peak in the diffracted light representing the colour. It is envisioned that the user captures the image of the holographic sensor and uploads the image to the app which executes automatically the image encryption and compression and, subsequently, transfers the output image to the colour for colour image processing. Further studies are required to establish the robustness of the proposed algorithm for real-world settings.

## Supporting information

S1 Fig Colour image database of six pH-sensitive holographic grating sensors (11 pH levels). (TIFF)
