# Supplementary figures and images for: Smartphone-based quantitative measurements on holographic sensors
Source: PLoS One. 2017 Nov 15;12(11):e0187467. doi: 10.1371/journal.pone.0187467 (PMC5687774; doi:10.1371/journal.pone.0187467)

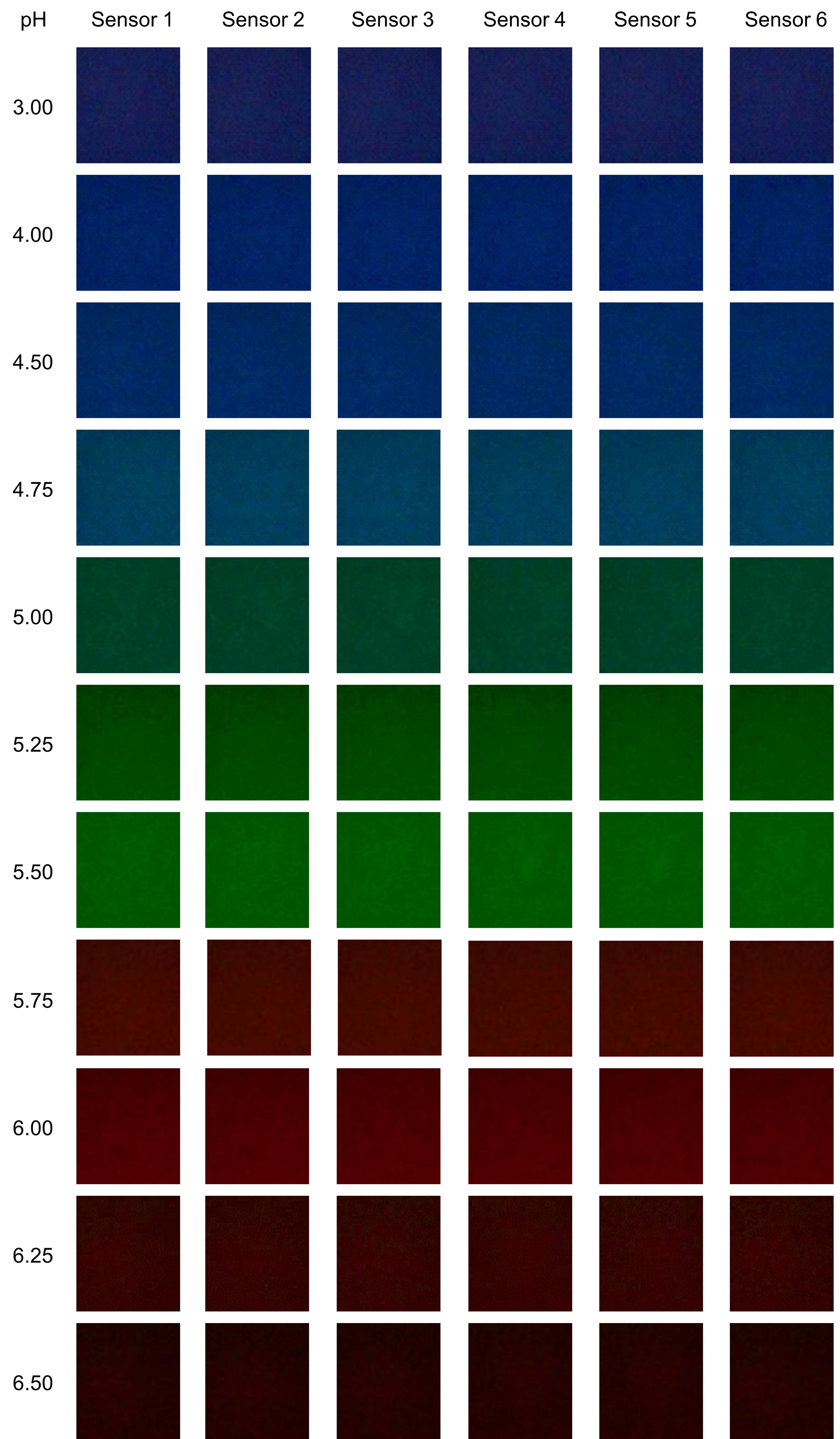

Supplement: S1 Fig — (TIFF) [file pone.0187467.s001.tiff]
